# scURL: uncertainty-sharpened representation learning for single-cell multi-omics clustering

**DOI:** 10.1093/bioinformatics/btag723

**Published:** 2026-09-29

**Authors:** Hongwei Liu, Donghui Yu, Wei Li, Tong Zhang, Ching-Yu Cheng, Huazhu Fu, Hong Liang, Shan Cong, Xiaohui Yao

**Affiliations:** College of Intelligent Systems Science and Engineering, Harbin Engineering University, Harbin, 150001, China; Qingdao Innovation and Development Center of Harbin Engineering University, Qingdao, 266000, China; Sanya Nanhai Innovation and Development Base of Harbin Engineering University, Sanya, 572024, China; College of Intelligent Systems Science and Engineering, Harbin Engineering University, Harbin, 150001, China; Qingdao Innovation and Development Center of Harbin Engineering University, Qingdao, 266000, China; College of Intelligent Systems Science and Engineering, Harbin Engineering University, Harbin, 150001, China; Qingdao Innovation and Development Center of Harbin Engineering University, Qingdao, 266000, China; Stem Cell and Regenerative Biology, Genome Institute of Singapore, A*STAR, Singapore, 138672, Singapore; Department of Ophthalmology, Yong Loo Lin School of Medicine, National University of Singapore, Singapore, 119228, Singapore; Institute of Advanced Intelligence and Computing (IAIC), A*STAR, Singapore, 138632, Singapore; College of Intelligent Systems Science and Engineering, Harbin Engineering University, Harbin, 150001, China; Sanya Nanhai Innovation and Development Base of Harbin Engineering University, Sanya, 572024, China; College of Intelligent Systems Science and Engineering, Harbin Engineering University, Harbin, 150001, China; Qingdao Innovation and Development Center of Harbin Engineering University, Qingdao, 266000, China; Sanya Nanhai Innovation and Development Base of Harbin Engineering University, Sanya, 572024, China; Stem Cell and Regenerative Biology, Genome Institute of Singapore, A*STAR, Singapore, 138672, Singapore; College of Intelligent Systems Science and Engineering, Harbin Engineering University, Harbin, 150001, China; Qingdao Innovation and Development Center of Harbin Engineering University, Qingdao, 266000, China; Sanya Nanhai Innovation and Development Base of Harbin Engineering University, Sanya, 572024, China; Department of Ophthalmology, Yong Loo Lin School of Medicine, National University of Singapore, Singapore, 119228, Singapore

## Abstract

**Motivation:**

Single-cell multi-omics clustering requires integrating complementary omics layers while accounting for their unequal reliability across individual cells. However, cell-wise reliability can vary substantially within each omics layer, causing different omics to provide reliable signals for some cells but ambiguous or noisy signals for others. This challenge is further complicated by omics-level heterogeneity, where differences in sparsity, signal distribution, and biological resolution may introduce cross-omics conflicts and bias the fused representation.

**Results:**

We present scURL, an uncertainty-sharpened representation learning framework for single-cell multi-omics clustering. scURL introduces representation uncertainty (RU) and establishes its connection to clustering generalization risk (GR), enabling the derivation of cell-wise omics weights for uncertainty-aware fusion (UAF). To support stable uncertainty estimation, scURL further introduces a multi-granular calibration module (MCM) that calibrates omics-specific representations from both cluster-level semantic and local-level structural perspectives before fusion. Experiments on ten datasets demonstrate that scURL outperforms existing clustering methods, remains robust under dropout noise, and identifies biologically meaningful cell populations, as supported by marker gene analysis.

**Availability and Implementation:**

Source code is available at https://github.com/Yaolab-fantastic/scURL.

## 1 Introduction

Single-cell multi-omics clustering aims to identify cellular populations by integrating complementary molecular layers that reflect different aspects of cellular state and regulation (Chen *et al.* 2022). By jointly modeling information from transcriptomic, epigenomic, proteomic, and other omics layers, multi-omics data provide a richer view of cellular heterogeneity than any single layer alone. However, these omics layers often differ in data characteristics, signal quality, and biological resolution, making it challenging to learn unified cell representations that preserve complementary information while limiting inconsistent or less informative signals (Zeng 2022, Qiu *et al.* 2024). This has led to growing interest in deep learning-based integration for single-cell multi-omics clustering.

Current approaches can be broadly categorized into two groups. One group aims to align different omics layers in the data or latent space (Hao *et al.* 2021, Cao and Gao 2022). Representative examples include projection-based methods using canonical correlation analysis [CCA (Cheng *et al.* 2022)], autoencoder-based joint embedding methods (Zuo *et al.* 2021, Ashuach *et al.* 2023), and graph-based approaches that align cross-omics cell graphs (Cao and Gao 2022). Although effective for constructing unified representations, these methods typically optimize a shared aligned space rather than explicitly modeling omics-specific reliability. Given that omics heterogeneity can yield uneven or conflicting clustering evidence, alignment alone may be insufficient to distinguish reliable signals from weak or inconsistent ones (Zeng 2022).

A second line of work integrates omics layers through fixed or adaptive fusion, ranging from direct concatenation (Gayoso *et al.* 2021, Lin *et al.* 2022) to clustering-consistency-based weighting (Eltager *et al.* 2022, Chen *et al.* 2024) and attention-based fusion (Ren *et al.* 2023, Hu *et al.* 2024). Rather than enforcing a shared space, these methods combine omics-specific embeddings by adjusting their relative contributions. However, existing fusion criteria are often global, heuristic, or learned without an explicit cell-level basis. Since omics quality and biological signal can vary across cells, such schemes may mix reliable and noisy signals indiscriminately, leading to unstable or suboptimal representations.

To address these limitations, we propose scURL, an uncertainty-sharpened representation learning framework for single-cell multi-omics clustering. scURL introduces representation uncertainty (RU) to quantify the ambiguity of each omics-specific embedding with respect to cluster boundaries, providing a cell-level measure of omics reliability. We further formulate uncertainty-aware fusion (UAF) from the perspective of clustering generalization risk (GR), which leads to fusion weights that are explicitly tied to the expected quality of the fused clustering representation (§2.1).

The effectiveness of RU-guided fusion, however, depends on whether RU can be estimated from well-structured and comparable omics-specific embeddings. Cross-omics conflicts in single-cell data may obscure cluster boundaries and distort the uncertainty derived from soft cluster assignments. To this end, scURL further introduces a multi-granular calibration module (MCM, §2.2.2) before fusion, which refines omics-specific embeddings by promoting cluster-level semantic consistency and neighborhood-level structural consistency. By calibrating embeddings, MCM provides a stronger basis for cell-wise reliability assessment and complements RU-guided fusion in learning robust clustering representations.

Our main contributions are summarized as follows:

- We introduce representation uncertainty (RU) to improve single-cell multi-omics representation learning for clustering. By quantifying the ambiguity of embeddings relative to cluster boundaries, RU can assess the quality of each omics layer at the cell level and thereby guide the dynamic fusion of different omics.
- We reformulate the RU-based fusion problem through the lens of clustering generalization risk (GR). Building on the GR upper-bound formulation, we derive a closed-form weighting rule that provides a principled motivation for uncertainty-sharpened fusion.
- We propose a multi-granular calibration module (MCM) to refine omics-specific embeddings. MCM promotes cluster-level semantic consistency and neighborhood-level structural consistency, providing a stronger basis for cell-wise RU estimation.
- Extensive experiments on ten datasets show that scURL learns effective cell representations and achieves superior performance.

## 2 Methods

We first establish the theoretical basis for uncertainty-sharpened multi-omics fusion by linking cell-wise representation uncertainty to clustering generalization risk in §2.1. Building on this formulation, we then present the scURL framework in §2.2 (Fig. 1), which combines encoding, multi-granular calibration, and uncertainty-guided fusion.

**Figure 1:**
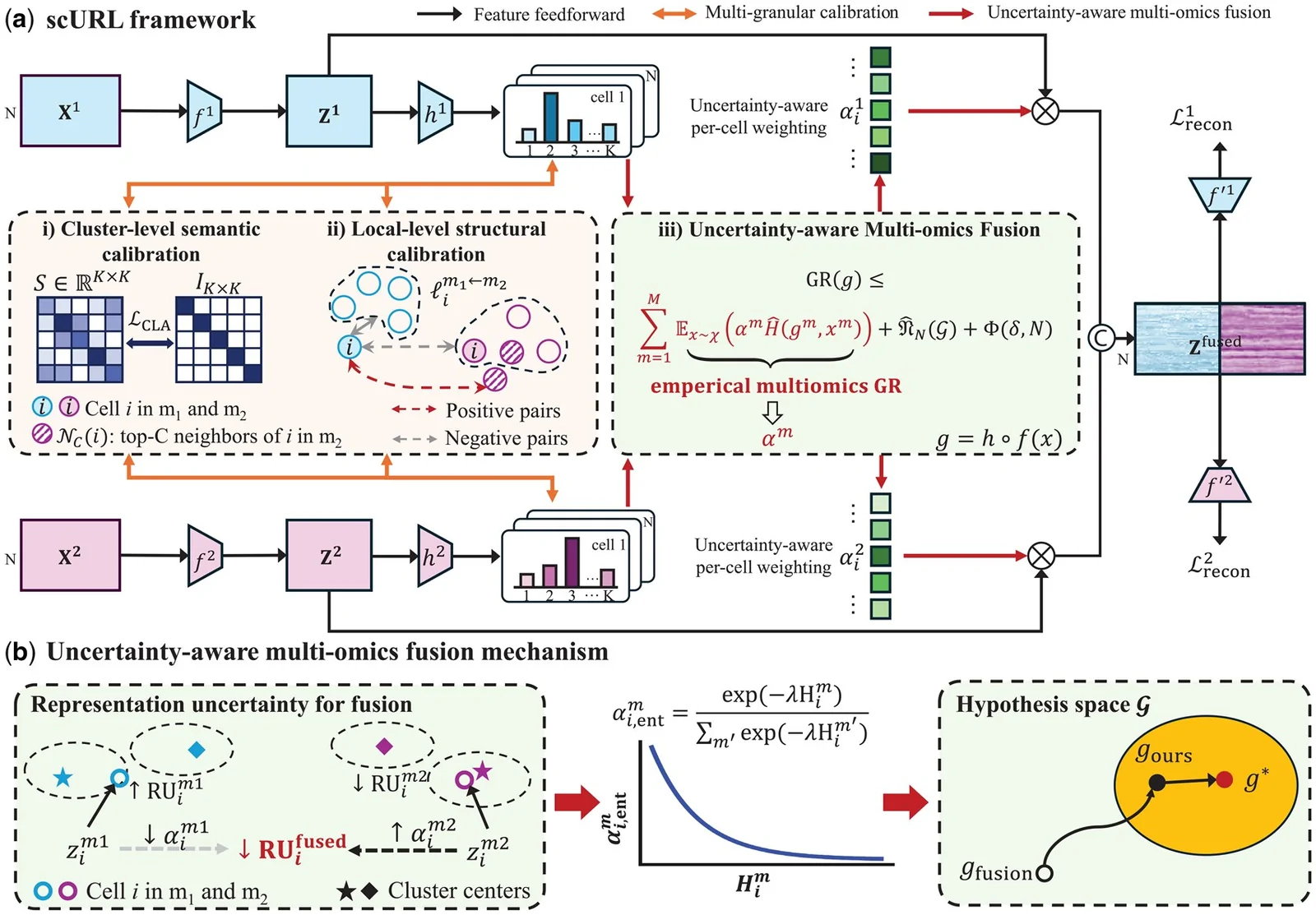
(a) Overview of scURL. i) *Cluster-level semantic calibration* enforces cross-omics semantic consistency by aligning cluster soft-assignment distributions; ii) *Local-level structural calibration* performs neighborhood-guided alignment by matching the soft labels of each cell to a randomly selected top-*C* cross-omics neighbor; iii) *Multi-omics clustering GR bound* links entropy-based representation uncertainty to generalization, enabling per-cell, uncertainty-aware omics weighting. (b) The mechanism of uncertainty-sharpened multi-omics fusion. It applies normalized exponential Gibbs weights based on negative entropy to generate fused embeddings, encouraging lower-uncertainty omics to contribute more strongly and yielding robust, better-separated representations.

### 2.1 Uncertainty-sharpened multi-omics fusion: theory

Let X denote the input space, where each cell $x=\{x^{1},\ldots ,x^{M}\}\in X$ contains measurements from *M* omics layers. For each omics *m*, an encoder $f^{m}:X^{m}\mapsto Z$ maps $x_{i}^{m}$ into latent representation $z_{i}^{m}=f^{m}(x_{i}^{m})$. The fused embedding of cell *i* is obtained by


(1) $$z_{i}^{\text{fused}}=\sum_{m=1}^{M}\alpha_{i}^{m}z_{i}^{m},\;\alpha_{i}^{m}\geq 0,\;\sum_{m=1}^{M}\alpha_{i}^{m}=1.$$

To characterize cluster structure, a clustering head $h:Z\;\mapsto \;\Delta^{K-1}$ produces the soft cluster assignment $p_{h}(\cdot |z_{i})=h(z_{i})$ over *K* clusters. Thus, the goal is to learn a robust model g=h°f(x) that yields discriminative cell representations for clustering.

The uncertainty of a representation reflects its ambiguity with respect to cluster boundaries. Entropy provides a natural characterization of RU (Wang *et al.* 2022). Given a model *g* and its predicted soft clustering distribution $p_{g}(\cdot |x)$, we therefore define the clustering surrogate loss as the entropy of the assignment distribution:


(2) $$\ell (g,x):=H(g,x)=H\left(p_{g}(\cdot |x)\right),$$

where H(·) is the entropy function.

The generalization risk of *g* is then defined as $\text{GR}(g)=E_{x∼X}[\ell (g,x)]$. In the multi-omics setting, where omics embeddings are fused with weights ${\left\{\alpha^{m}\right\}}_{m=1}^{M}$, we obtain the following bound:

Theorem 1 (Multi-omics clustering generalization risk bound). Let G denote the hypothesis set, with each hypothesis being a mapping $g:X\to \Delta^{K-1}$. For input $x=(x^{1},\ldots ,x^{M})$, let $g^{m}\in G$ denote the hypothesis corresponding to omics m. Then, with probability 1−δ,

(3) $$\text{GR}(g)\leq \sum_{m=1}^{M}E\left[\alpha^{m}\hat{H}(g^{m},x^{m})\right]+{\hat{R}}_{N}(G)+\sqrt{\frac{\text{ln}(2/\delta )}{2N}}.$$

Here, $\hat{H}$ denotes the empirical entropy loss and ${\hat{R}}_{N}(G)$ denotes the empirical Rademacher complexity of G (Li and Liu 2021). The assumptions and proof are provided in the Supp Material S1.

Since the model complexity and confidence terms remain invariant under a given architecture, tightening the upper bound of multi-omics GR reduces to minimizing the expected empirical risk while preventing omics collapse. To achieve this, we formulate an optimization problem that assigns per-cell omics weights as


(4) $$\underset{\alpha_{i}\in \Delta^{M-1}}{\text{min}}\;\sum_{m=1}^{M}\alpha_{i}^{m}H_{i}^{m}+\frac{1}{\lambda }\sum_{m=1}^{M}\alpha_{i}^{m}\;\text{log}\;\alpha_{i}^{m},\;\lambda >0,$$

where $H_{i}^{m}$ is the entropy and $\Delta^{M-1}$ is the (M−1)-dimensional probability simplex. The regularizer prevents weight collapse. This convex program admits a unique Gibbs-type closed-form solution


(5) $$\alpha_{i,\text{ent}}^{m}=\frac{\;\text{exp}(-\lambda H_{i}^{m})}{\sum_{m^{\prime }=1}^{M}\;\text{exp}\;(-\lambda H_{i}^{m^{\prime }})}.$$

Thus, representations with smaller RU receive larger weights, consistent with the intuition that clearer cluster boundaries provide more reliable signals for clustering.

Algorithm 1 Training procedure of the scURL framework

**Input:** Multi-omics dataset $D={\left\{{\left\{x_{i}^{m}\right\}}_{i=1}^{N}\right\}}_{m=1}^{M}$; number of target clusters *K*; number of top neighbors *C* in LSC; pretraining and total epochs $T_{p},T_{e}$.

**Output:** Parameters for network g(·)=h°f(·).

1: **Initialize** parameters for encoders ${\left\{f^{m}\right\}}_{m=1}^{M}$, decoders ${\left\{f^{\prime m}\right\}}_{m=1}^{M}$, and clustering head $h^{m}:Z\to \Delta^{K-1}$.

2: Pretrain ${\left\{f^{m},f^{\prime m}\right\}}_{m=1}^{M}$ for $T_{p}$ epochs using $L_{\text{recon}}$.

3: **for**  t=1  **to**  $T_{e}$  **do** 

4:  **for** each modality m∈{1,…,M}  **do** 

5:   $z_{i}^{m}\leftarrow$ feed $x_{i}^{m}$ to each encoder $f^{m}$

6:   $p_{i}^{m}\leftarrow h^{m}(z_{i}^{m})$  ▹  Eq. 7

7:  **end for** 

8:  $L_{\text{CLC}}\leftarrow$ cluster-level semantic calibration ▹  Eqs. 8–9

9:  $L_{\text{LSC}}\leftarrow$ local-level structural calibration ▹  Eqs. 10–11

10:  Obtain $H_{i}^{m}$ and derive $\alpha_{i}^{m}$  ▹  Eqs. 2, 5

11:  $z_{i}^{\text{fused}}\leftarrow \sum_{m=1}^{M}\alpha_{i}^{m}z_{i}^{m}$

12:  **for** each omics m∈{1,…,M}  **do** 

13:   Decoding $z_{i}^{\text{fused}}$ with $f^{\prime m}$ and reconstruct ▹  Eq. 6

14:  **end for** 

15:  Compute L and update $\left\{f,f^{\prime },h\right\}$ by one Adam step ▹  Eq. 13

16: **end for** 

### 2.2 scURL architecture

Figure 1 and Algorithm 1 summarize the scURL architecture. Given multi-omics measurements of each cell, scURL first learns omics-specific latent representations and soft cluster assignments. These representations are then refined by a multi-granular calibration module [MCM, Fig. 1a(i–ii)], which promotes cluster-level semantic consistency and neighborhood-level structural consistency across omics. Finally, RU-derived cell-wise weights are used to fuse omics-specific representations for clustering (Fig. 1a(iii) and b).

#### 2.2.1 Omics-specific embedding and soft assignment

We employ an omics-specific encoder-decoder backbone to learn latent cell representations. For each omics *m*, the encoder $f^{m}$ maps $x_{i}^{m}$ to $z_{i}^{m}$, and a data-type-specific decoder reconstructs $x_{i}^{m}$ using ZINB for scRNA-seq/scADT-seq and Bernoulli for binary-like scATAC-seq. The overall reconstruction loss is:


(6) $$L_{\text{recon}}=\sum_{m\in M_{\text{ZINB}}}L_{\text{ZINB}}^{m}+\sum_{m\in M_{\text{Ber}}}L_{\text{Ber}}^{m}.$$

To capture the clustering structure of each omics-specific latent space, we further equip each omics with a clustering head $h^{m}:Z\to \Delta^{K-1}$, which produces the soft cluster assignment:


(7) $$p_{i}^{m}=h^{m}(z_{i}^{m})=\left[p_{i1}^{m},\ldots ,p_{iK}^{m}\right],$$

where $p_{ik}^{m}$ denotes the probability that cell *i* belongs to cluster *k* under omics *m*. These assignments are subsequently used to quantify cell-wise RU and to guide multi-granular calibration across omics.

#### 2.2.2 Multi-granular calibration module (MCM)

We then introduce a multi-granular calibration module (MCM) to refine omics-specific representations through semantic calibration across clusters and structural calibration across local neighborhoods.

*Cluster-level semantic calibration*. Given the soft assignments in Eq. (7), let $p_{k}^{m}={\left[p_{1k}^{m},\ldots ,p_{Nk}^{m}\right]}^{⊤}$ denote the assignment distribution of all cells to cluster *k* under omics *m*. To encourage clusters with matched semantics to be aligned across omics, we define the contrastive loss between two omics $m_{1}$ and $m_{2}$ as


(8) $$\ell_{\text{CLC}}(m_{1},m_{2})=-\frac{1}{K}\sum_{k=1}^{K}\;\text{log}\;\frac{\;\text{exp}\;\left(s(p_{k}^{m_{1}},p_{k}^{m_{2}})/\tau \right)}{\sum_{j=1,j\neq k}^{K}\;\text{exp}\;\left(s(p_{k}^{m_{1}},p_{j}^{m_{1}})/\tau \right)+\sum_{j=1}^{K}\;\text{exp}\;\left(s(p_{k}^{m_{1}},p_{j}^{m_{2}})/\tau \right)},$$

where s(·,·) is cosine similarity and τ is a temperature parameter.

The overall cluster-level calibration loss combines the contrastive alignment term over all omics pairs and a balancing regularizer that avoids degenerate cluster usage:


(9) $$L_{\text{CLC}}=\sum_{\overset{m_{1},m_{2}=1}{m_{1}\neq m_{2}}}^{M}\ell_{\text{CLC}}(m_{1},m_{2})+\sum_{m=1}^{M}\sum_{k=1}^{K}q_{k}^{m}\;\text{log}\;q_{k}^{m}.$$

The first term aligns cluster semantics across omics, while the second term encourages balanced cluster usage. Here, $q_{k}^{m}$ denotes the average assignment probability of cells in omics *m* to cluster *k*.

*Local-level structural calibration*. While CLC aligns global cluster semantics, local neighborhood structures may still differ across omics. Here, we introduce local-level structural calibration (LSC), which aligns each cell with its cross-omics neighbors rather than enforcing strict one-to-one matching. For cell *i* in omics $m_{1}$, we randomly select a cell $j\in N_{C}^{m_{2}}(i)$ from the top-*C* cells in $m_{2}$ identified at each epoch by cosine similarity between their embeddings, excluding the paired cells from other omics. The directional LSC loss is defined as:


(10) $$\ell_{i}^{m_{1}\leftarrow m_{2}}=-\text{log}\;\frac{\;\text{exp}\;\left(s(p_{i}^{m_{1}},p_{j}^{m_{2}})/\tau \right)}{\sum_{n\neq j}\;\text{exp}\;\left(s(p_{i}^{m_{1}},p_{n}^{m_{2}})/\tau \right)+\sum_{n\neq i}\;\text{exp}\;\left(s(p_{i}^{m_{1}},p_{n}^{m_{1}})/\tau \right)}.$$

The numerator aligns the selected cross-omics neighbor, while the denominator contrasts cross-omics and intra-omics negatives.

The reverse-direction loss is computed symmetrically, and the overall LSC loss is:


(11) $$L_{\text{LSC}}=\frac{1}{2N}\sum_{i=1}^{N}\left(\ell_{i}^{m_{1}\leftarrow m_{2}}+\ell_{i}^{m_{2}\leftarrow m_{1}}\right)-\frac{1}{N}\sum_{i=1}^{N}\;\text{log}\;\left({\left(p_{i}^{m_{1}}\right)}^{T}p_{i}^{m_{2}}\right),$$

where the second term promotes cross-omics consistency.

#### 2.2.3 Uncertainty-aware multi-omics fusion

As discussed in §2.1, multi-omics fusion aims to adaptively adjust the contribution of different omics for each cell. According to Eq. (5), scURL assigns larger weights to lower-uncertainty omics, allowing more reliable signals to contribute more to the fused embedding:


(12) $$z_{i}^{\text{fused}}=\sum_{m=1}^{M}\alpha_{i,\text{ent}}^{m}z_{i}^{m},$$

where $\alpha_{i,\text{ent}}^{m}$ is the entropy-based weight of omics *m* for cell *i*.

### 2.3 Overall optimization and evaluation

Since the fusion weights are dynamically computed at each training iteration rather than introduced as additional trainable parameters, the overall objective of the model is expressed as:


(13) $$L=\eta_{1}L_{\text{recon}}+\eta_{2}L_{\text{CLC}}+\eta_{3}L_{\text{LSC}},$$

with $\eta_{1}$, $\eta_{2}$, and $\eta_{3}$ used to adjust the corresponding loss terms. Finally, *k*-means is applied to the fused representations $Z^{\text{fused}}$ after training to examine the semantic structure captured by scURL.

## 3 Experiments

### 3.1 Datasets and experimental settings

*Single-cell multi-omics datasets*. We used ten publicly available single-cell multi-omics datasets covering scRNA-seq, scATAC-seq, and scADT-seq. Details are summarized in Table 1.

**Table 1:** Overview of datasets used in the experiments.

Datasets	No. of cells	No. of features			No. of cell types
		scRNA	scATAC	scADT	
MA-53 (Ma *et al.* 2020)	5692	21 478	340 341	–	22
MA-54 (Ma *et al.* 2020)	10 709	21 478	340 341	–	22
MA-55 (Ma *et al.* 2020)	9903	21 478	340 341	–	22
MA-56 (Ma *et al.* 2020)	5927	21 478	340 341	–	22
Human cell line mixture (Chen *et al.* 2019)	1047	18 666	136 771	–	4
Mouse brain	3293	21 127	428 041	–	19
Specter PBMC (Ringeling *et al.* 2021)	3762	3000	–	49	16
BREM-SC PBMC (Wang *et al.* 2020)	1182	33 538	–	10	6
BMNC (Hu *et al.* 2024)	30 672	17 009	–	25	27
SLN111 (Hu *et al.* 2024)	16 828	13 553	–	112	35

*Benchmark methods*. We compared scURL with nine representative methods, including scDRMAE (Zhang *et al.* 2024), scEMC (Hu *et al.* 2024), scMCs (Ren *et al.* 2023), MoClust (Yuan *et al.* 2023), DealMVC (Yang *et al.* 2023), CVCL (Chen *et al.* 2023), scMVC (Wu *et al.* 2024), MCMC (Bian *et al.* 2025), and MGCMVC (Ju *et al.* 2025). All methods used their recommended hyperparameter settings.

*Implementation*. scURL was implemented in PyTorch and evaluated on a single NVIDIA RTX 3090 GPU, with 100 epochs of encoder pre-training followed by 500 epochs of end-to-end training. All models were optimized with Adam at a learning rate of $1\times 10^{-3}$.

*Evaluation metrics*. Clustering performance was evaluated using adjusted Rand index (ARI), normalized mutual information (NMI), and adjusted mutual information (AMI, see Supplementary Results).

### 3.2 scURL outperforms the state of the art

Tables 2 and Supplementary Table S1 report the comparison results on ten benchmark datasets. Overall, scURL achieves superior performance across most evaluation metrics and datasets, highlighting its effectiveness and robustness for sc-multi-omics clustering. Among sc-multi-omics methods, scEMC and scDRMAE perform well on some datasets but are less stable across benchmarks, suggesting that reconstruction- or attention-based objectives alone may not be sufficient to handle strong cross-omics heterogeneity.

**Table 2:** Comparison of clustering performance on ten datasets.

Method	MA-53		MA-54		MA-55		MA-56		Human cell line mixture		Mouse brain		Specter PBMC		BREM-SC PBMC		BMNC		SLN111	
	ARI	NMI	ARI	NMI	ARI	NMI	ARI	NMI	ARI	NMI	ARI	NMI	ARI	NMI	ARI	NMI	ARI	NMI	ARI	NMI
DealMVC	0.1483	0.3398	0.1145	0.3001	0.1197	0.2881	0.1166	0.3039	0.8472	0.7798	0.1012	0.2528	0.4864	0.6197	0.2834	0.4604	0.6341	0.7535	0.3848	0.5474
scDRMAE	0.4539	0.6631	0.3273	0.5652	0.3184	0.5753	0.3080	0.5708	0.9574	**0.9403**	0.4423	0.6485	0.5093	0.6820	0.9215	0.9148	0.5290	0.7437	0.2294	0.5622
scEMC	0.0333	0.1836	0.0124	0.0454	0.0164	0.0480	0.0441	0.1509	0.7511	0.7529	0.2205	0.4780	0.6026	**0.7333**	0.7993	0.8613	0.4509	0.6727	0.4592	**0.6128**
scMCs	0.3051	0.3944	0.2609	0.3117	0.2771	0.3408	0.2949	0.3465	0.9413	0.9017	0.2575	0.3253	0.2052	0.2813	0.5558	0.6399	0.2217	0.4319	0.0864	0.3005
MoClust	0.2938	0.4658	0.3748	0.4953	0.3223	0.4772	0.2647	0.3943	0.0323	0.0545	0.1109	0.2170	0.4189	0.5900	0.4682	0.6170	0.4633	0.6417	0.2916	0.4645
CVCL	0.1125	0.2278	0.0951	0.2438	0.0766	0.2229	0.1401	0.2629	0.4931	0.4881	0.0446	0.1574	0.4448	0.6393	–	–	0.1021	0.2454	0.0415	0.1471
scMVC	0.2094	0.4442	0.1241	0.3132	0.1191	0.3086	0.1357	0.3514	0.2587	0.3564	0.2999	0.5175	0.4590	0.6084	0.6847	0.8686	0.4929	0.7487	0.1576	0.4980
MCMC	0.1744	0.3494	0.1571	0.3461	0.1361	0.3324	0.2204	0.4204	0.3837	0.3786	0.1422	0.2964	0.4956	0.6193	0.4197	0.6614	0.2486	0.3808	0.2538	0.5473
MGCMVC	0.1901	0.3622	0.1868	0.2875	0.1934	0.3514	0.1951	0.3253	0.6759	0.6194	0.1151	0.2863	0.4613	0.6010	0.9166	0.8662	0.6070	0.7598	0.3507	0.5039
scURL	**0.6005**	**0.7433**	**0.3762**	**0.5839**	**0.3902**	**0.5914**	**0.4007**	**0.6056**	**0.9660**	0.9359	**0.5361**	**0.6746**	**0.6138**	0.6909	**0.9601**	**0.9232**	**0.6778**	**0.7863**	**0.4988**	0.6033

The best is in bold, and the second-best is underlined.

“–” Denotes that no results are available for the method on the corresponding dataset.

General multi-view clustering methods are less competitive, likely because they do not explicitly model single-cell noise and omics-specific biases. In contrast, scURL reduces cross-omics discrepancies through multi-granular alignment and adaptively adjusts omics contributions according to cell-wise representation uncertainty, producing more discriminative cell representations. As shown in Fig. 2, scURL yields compact and relatively well-separated manifolds, consistent with the quantitative results in Table 2.

**Figure 2:**
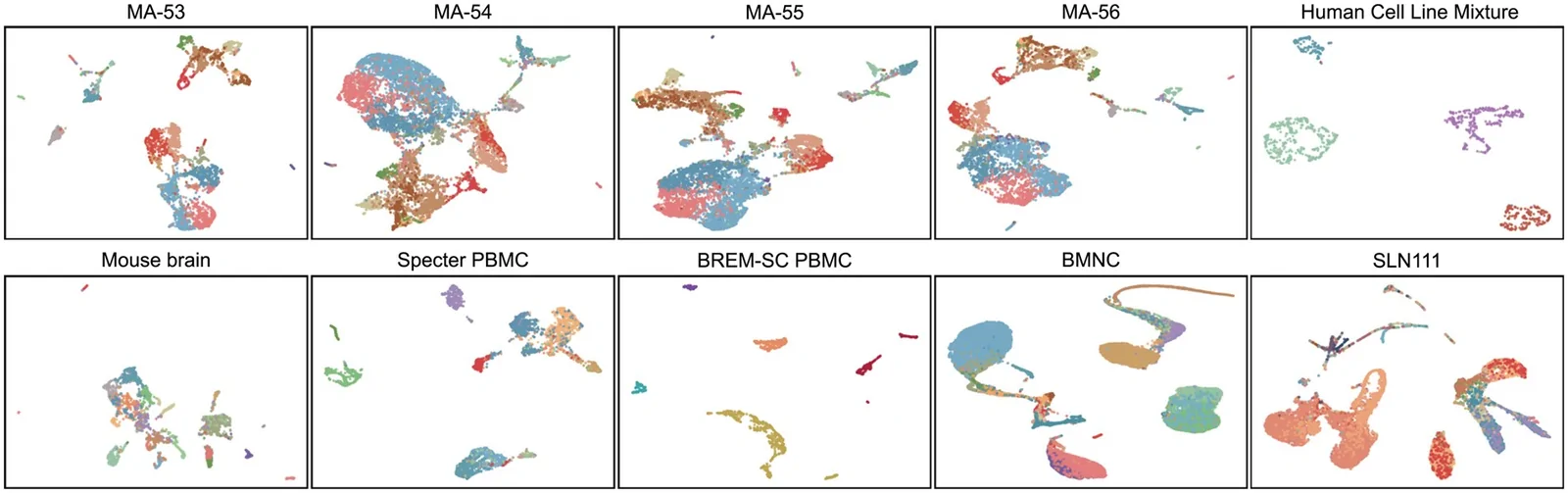
UMAP projections of learned cell representations across ten datasets. Points are colored by cell-type annotations.

### 3.3 Ablation analysis

We evaluated three scURL variants: the encoder–decoder backbone alone, with MCM, and with UAF. As shown in Table 3 and Table S2, both MCM and UAF improve clustering performance, while the complete model performs best, indicating their complementary roles in learning clustering-discriminative cell representations.

**Table 3:** Ablation studies of the key components.

MCM	UAF	MA-53		MA-54		MA-55		MA-56		Human cell line		Mouse brain		Specter PBMC		BREM-SC PBMC		BMNC		SLN111	
		ARI	NMI	ARI	NMI	ARI	NMI	ARI	NMI	ARI	NMI	ARI	NMI	ARI	NMI	ARI	NMI	ARI	NMI	ARI	NMI
×	×	0.4960	0.6960	0.2895	0.5400	0.3075	0.5570	0.3000	0.5600	0.8845	0.8550	0.3980	0.5975	0.5527	0.6703	0.7197	0.8113	0.5716	0.7341	0.3760	0.5456
✓	×	0.5922	0.7423	0.3648	0.5734	0.3830	0.5890	0.3760	0.6016	0.9363	0.8940	0.4246	0.6117	0.5920	0.6770	0.9298	0.8926	0.6225	0.7398	0.4027	0.5680
×	✓	0.5824	0.7358	0.3628	0.5779	0.3690	0.5840	0.3783	0.5979	0.9237	0.9022	0.4025	0.6064	0.5874	0.6771	0.9052	0.8776	0.6395	0.7734	0.4419	0.5913
✓	✓	**0.6005**	**0.7433**	**0.3762**	**0.5839**	**0.3902**	**0.5914**	**0.4007**	**0.6056**	**0.9660**	**0.9359**	**0.5361**	**0.6746**	**0.6138**	**0.6909**	**0.9601**	**0.9232**	**0.6778**	**0.7863**	**0.4988**	**0.6033**

We further evaluated the local structural calibration module by comparing single-omics and fused representations with and without it (Fig. 3). The LSC improves weaker ATAC representations in RNA-ATAC datasets and also benefits RNA, indicating bidirectional cross-omics information exchange. Similar gains are observed in RNA-ADT datasets and are further reflected in the fused embeddings, suggesting that local calibration helps strengthen cell population structure.

**Figure 3:**
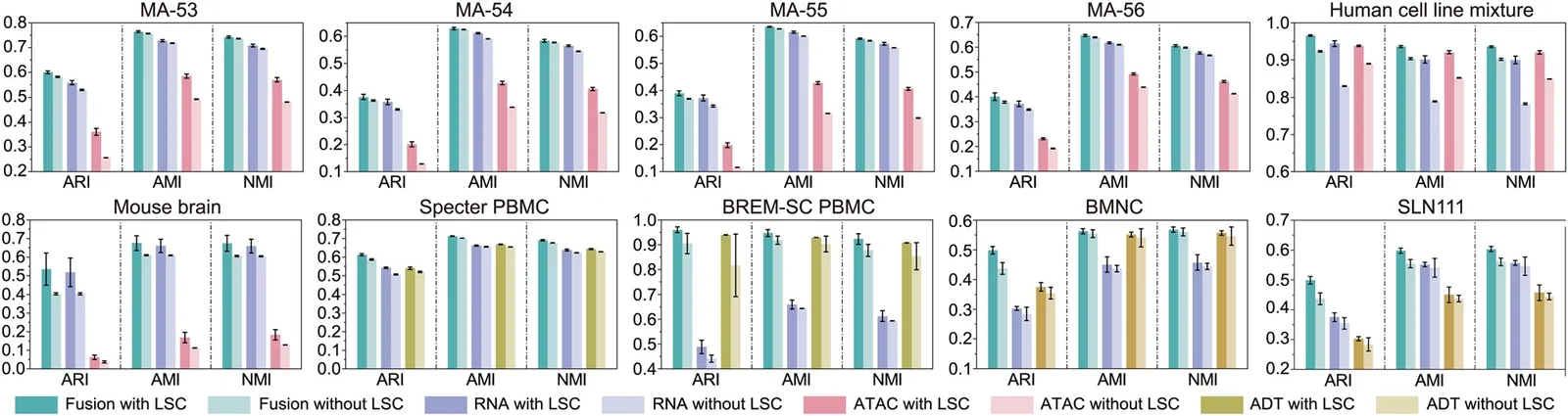
Clustering performance of omics-specific and fused representations with and without LSC across ten datasets.

### 3.4 Robustness and sensitivity analysis

We assessed robustness by randomly masking entries across the preprocessed feature matrix and retraining all methods. From Fig. 4, scURL consistently maintains the highest ARI and shows the most gradual performance decline as dropout increases.

**Figure 4:**
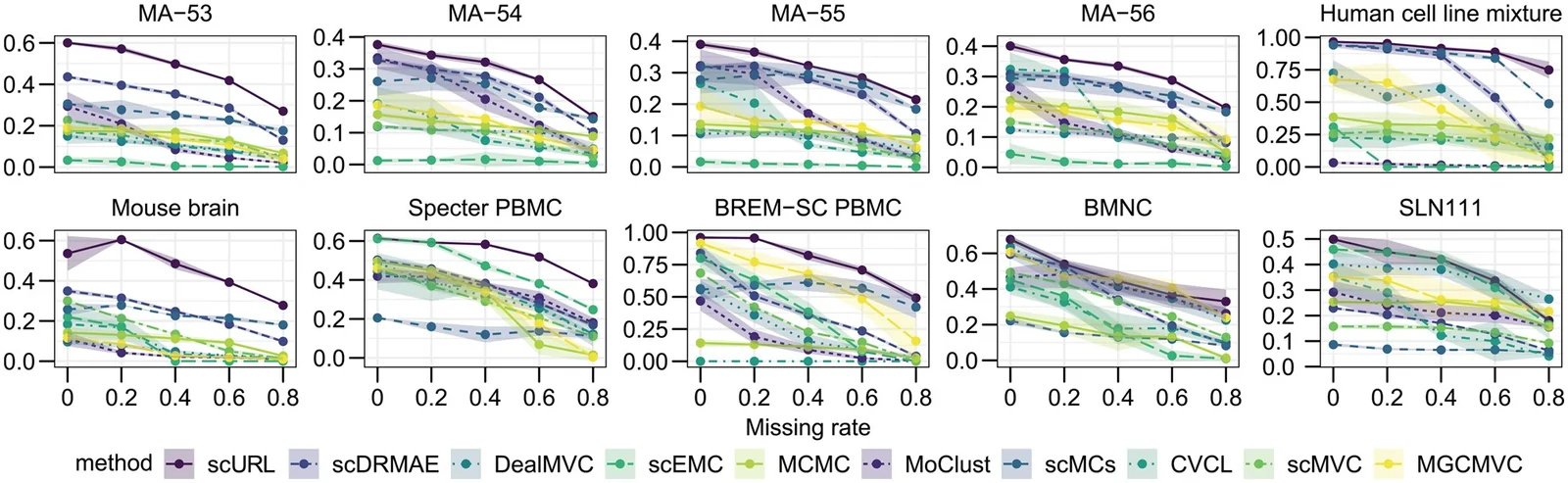
ARI comparison under increasing dropout ratios across ten datasets.

We evaluated sensitivity to the LSC neighborhood size *C* and the fusion sharpness parameter λ. Supplementary Figs. S1 and S2 show that scURL remains stable across *C* and λ. Since C=1 removes neighborhood information and is generally suboptimal, we used C=10 in all experiments. Cell-wise weight distributions are in Fig. S3.

### 3.5 Biological interpretation of identified cell populations

We performed marker gene analysis to examine the biological identity of the clusters identified by scURL. Genes within each predicted cluster were ranked using the Wilcoxon rank-sum test, and the top markers and expression patterns are shown in Fig. 5. The top-ranked markers match canonical immune cell programs, including *TRAC* and *LTB* for CD4${\;}^{+}$ T cells, *GNLY*, *GZMB*, and *NKG7* for natural killer cells, *CD74*, *CD79A*, and *CD79B* for B cells, and *S100A8* and *S100A9* for classical CD14${\;}^{+}$ monocytes (Ng *et al.* 2020, Shou *et al.* 2021). These results support the biological relevance of the identified clusters.

**Figure 5:**
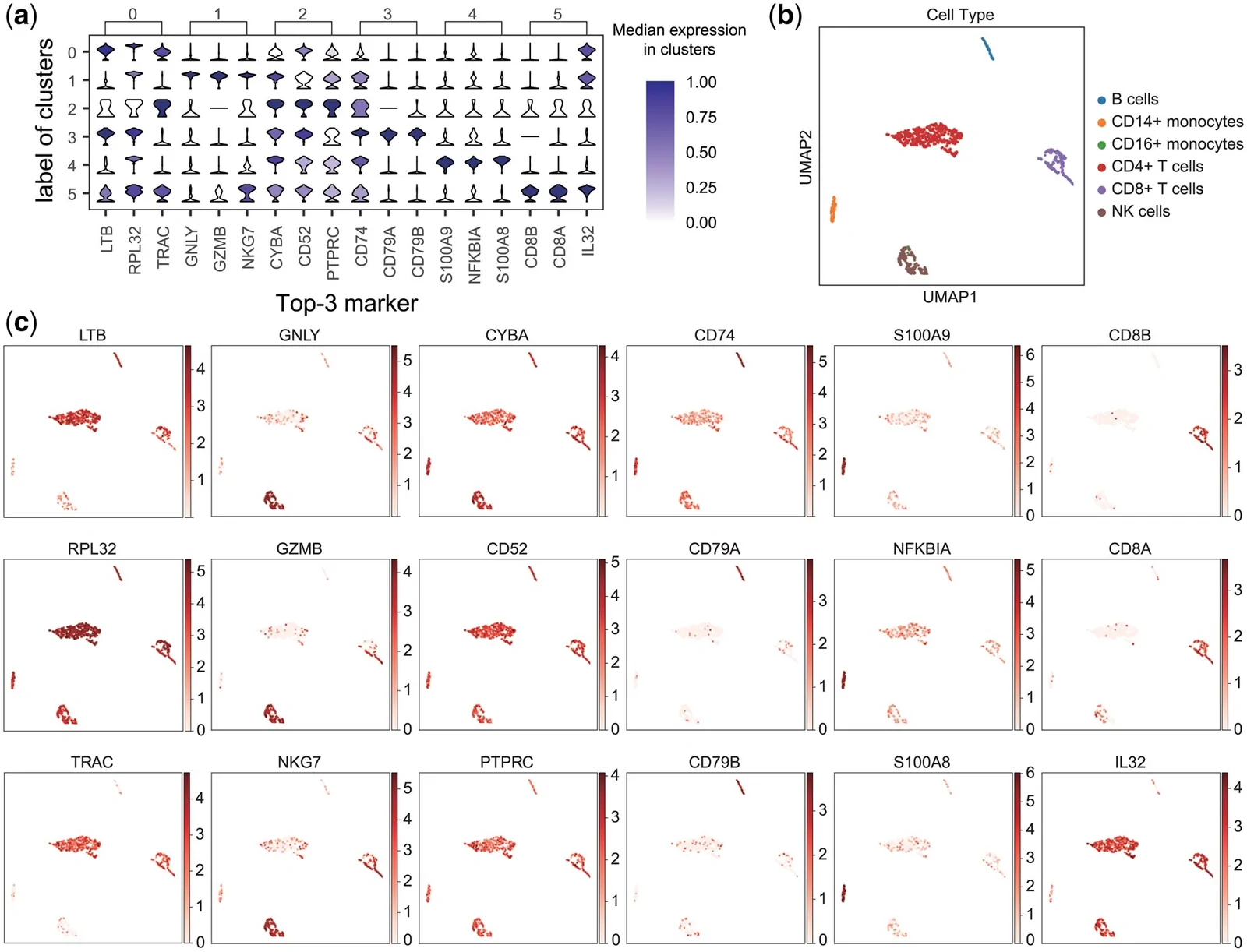
Marker gene identification and visualization across different cell types. (a) Stacked violin plot showing the expression distribution of the top three differentially expressed genes within each predicted cluster on the BREM-SC PBMC dataset. (b) UMAP plot showing the ground truth distribution of major immune cell types. (c) UMAP visualization of the expression patterns of the identified marker genes across different clusters.

The analysis also shows that the CD16${\;}^{+}$ monocyte cluster has weaker marker specificity. Its top-ranked genes, including *CYBA*, *CD52*, and *PTPRC*, mainly reflect generic myeloid or leukocyte programs rather than CD16${\;}^{+}$-specific markers, consistent with its limited separation from other monocyte subsets in Fig. 5.

## 4 Discussion and conclusion

In this work, we propose scURL, an uncertainty-sharpened representation learning framework for single-cell multi-omics clustering. The framework builds on a theoretical formulation that links representation uncertainty to clustering generalization risk. This formulation provides an upper-bound-inspired basis for deriving entropy-based fusion weights. This provides a theoretically motivated way to assess cell-level reliability and guide adaptive multi-omics fusion. To further improve the quality of uncertainty estimation, a multi-granular calibration module is introduced to reduce omics conflicts and refine omics-specific embeddings at cluster and local-neighborhood levels. Extensive experiments show that scURL produces stable and expressive representations that preserve meaningful cellular structure and remain robust under perturbation.

Future work will extend scURL to unpaired or partially observed omics, broader omics layers such as spatial omics, and semi-supervised or transfer settings for scalable and interpretable analysis.

## Supplementary Material

btag723_Supplementary_Data

## Data Availability

The data underlying this study are publicly available from previously published sources and open repositories. All single-cell multi-omics datasets used in this work can be accessed through the Gene Expression Omnibus (https://www.ncbi.nlm.nih.gov/geo/) and the 10x Genomics Data Portal (https://www.10xgenomics.com/resources/datasets). Detailed dataset information and corresponding references are provided in the manuscript.
