# Supplementary material for: scURL: uncertainty-sharpened representation learning for single-cell multi-omics clustering
Source: Bioinformatics. 2026 Sep 29;42(10):btag723. doi: 10.1093/bioinformatics/btag723 (PMC13661260; doi:10.1093/bioinformatics/btag723)
Supplement: Supplementary file 1 [file btag723_supplementary_data.pdf]

Supplementary Material for “scURL: Uncertainty-Sharpened Representation Learning for Single-cell Multi-omics Clustering”

Hongwei Liu, Donghui Yu, Wei Li, Tong Zhang, Ching-Yu Cheng, Huazhu Fu, Hong Liang, Shan Cong*, Xiaohui Yao*

S1 Theoretical analysis of uncertainty-sharpened multi-omics fusion

S1.1 Entropy as a surrogate for representation uncertainty

Let $\mathcal{X}$ denote the input space, where each cell $x = \{x^1, \ldots, x^M\} \in \mathcal{X}$ contains measurements from M omics layers. For a clustering model $g = h \circ f$, the clustering head outputs a soft cluster assignment distribution $p_g(\cdot \mid x) \in \Delta^{K-1}$ over K clusters. In scURL, representation uncertainty (RU) is measured by the Shannon entropy of the soft assignment distribution,

$$\ell(g, x) := H(g, x) = H(p_g(\cdot \mid x)) . \tag{S1}$$

A concentrated assignment indicates clear cluster evidence and lower uncertainty, whereas a diffuse assignment indicates ambiguous cluster evidence and lower reliability.

We provide an information-theoretic justification for this entropy-based RU measure. Let $Y \in \{1, \ldots, K\}$ denote the latent cluster label with conditional distribution $P^*(\cdot \mid x)$. Given the argmax predictor $\hat{Y}_g(x) = \arg\max_c p_g(c \mid x)$, define the per-cell misclustering probability as

$$\varepsilon(g, x) = \Pr_{Y \sim P^*(\cdot|x)} \left[\hat{Y}_g(x) \neq Y\right] . \tag{S2}$$

Assume approximate calibration between the latent cluster distribution and the predicted soft assignment,

$$\mathrm{TV}(P^*(\cdot \mid x), p_g(\cdot \mid x)) \leq \bar{\delta}, \quad \bar{\delta} \in [0, 1), \tag{S3}$$

where $\mathrm{TV}(\cdot, \cdot)$ denotes total variation distance. Define

$$\phi(\bar{\delta}) = h(\bar{\delta}) + \bar{\delta} \log(K - 1), \quad h(t) = -t \log t - (1 - t) \log(1 - t). \tag{S4}$$

Lemma 1 (Entropy-calibrated lower bound) *For $K \geq 2$, under Eq.* (S3)*, the per-cell misclustering probability satisfies*

$$\varepsilon(g, x) \geq \frac{1}{\log K} H(p_g(\cdot \mid x)) - \frac{\phi(\bar{\delta}) + \log 2}{\log K}. \tag{S5}$$

Proof. By Fano’s inequality,

$$H(Y \mid x) \leq h(\varepsilon(g, x)) + \varepsilon(g, x) \log(K - 1). \tag{S6}$$

Using $h(\varepsilon) \le \log 2$ and $\log(K-1) \le \log K$ gives

$$\varepsilon(g, x) \ge \frac{H(Y \mid x) - \log 2}{\log K}. \tag{S7}$$

The calibration assumption in Eq. (S3), together with the Fannes–Audenaert entropy continuity bound, gives

$$|H(P^*(\cdot \mid x)) - H(p_g(\cdot \mid x))| \le \phi(\bar{\delta}). \tag{S8}$$

Substituting this inequality into Eq. (S7) yields Eq. (S5). □

Eq. (S5) shows that higher assignment entropy increases the lower bound of the misclustering probability up to a calibration-dependent slack term. This supports the use of soft-assignment entropy as a label-free surrogate for RU in single-cell multi-omics clustering.

S1.2 Assumptions for the multi-omics clustering GR bound

The general model $g = h \circ f$ introduced in the main text represents a generic clustering hypothesis. In the multi-omics setting, it is instantiated for each omics layer as $g^m = h^m \circ f^m \in \mathcal{G}$, where f^m and h^m are the corresponding encoder and clustering head. For each cell i, let

$$H_i^m = H(p_{g^m}(\cdot \mid x_i^m)) \tag{S9}$$

denote the entropy-based RU under omics m. The modality-specific entropy losses are combined using cell-wise weights $\alpha_i^m \ge 0$ with $\sum_{m=1}^{M} \alpha_i^m = 1$ in the subsequent GR formulation.

We use the following assumptions.

1. The entropy loss is bounded for all $g \in \mathcal{G}$ and all x, i.e., $0 \le H(p_g(\cdot \mid x)) \le \log K$. Equivalently, the entropy can be normalized by $\log K$ to lie in $[0, 1]$.

2. The hypothesis class $\mathcal{G}$ has finite empirical Rademacher complexity $\hat{\mathfrak{R}}_N(\mathcal{G})$ under the entropy-loss composition.

3. The cell-wise omics weights lie in the probability simplex. Therefore, the weighted entropy loss is a convex combination of omics-specific entropy losses.

4. The architecture and training sample size are fixed when deriving the cell-wise fusion rule, so the complexity and confidence terms in the GR bound are not directly optimized by the fusion weights.

S1.3 Proof of the multi-omics clustering GR bound

Define the weighted empirical entropy risk as

$$\widehat{\mathrm{GR}}_\alpha(g) = \frac{1}{N} \sum_{i=1}^{N} \sum_{m=1}^{M} \alpha_i^m H(p_{g^m}(\cdot \mid x_i^m)) \,. \tag{S10}$$

Theorem 1 (Multi-omics clustering generalization risk bound) *Let $\mathcal{G}$ denote the hypothesis set, with each hypothesis being a mapping $g : \mathcal{X} \to \Delta^{K-1}$. For input $x = (x^1, \ldots, x^M)$, let $g^m \in \mathcal{G}$ denote the hypothesis corresponding to omics m. Then, with probability at least $1 - \delta$,*

$$\mathrm{GR}(g) \le \sum_{m=1}^{M} \mathbb{E}\Big[\alpha^m \hat{H}(g^m, x^m)\Big] + \hat{\mathfrak{R}}_N(\mathcal{G}) + \sqrt{\frac{\ln(2/\delta)}{2N}}. \tag{S11}$$

Here, $\hat{H}$ denotes the empirical entropy loss. For simplicity, the bound is written for a loss normalized to $[0,1]$. If the standard unnormalized entropy $H(p) = -\sum_{k=1}^{K} p_k \log p_k$ is used, the concentration term should be multiplied by the entropy range $\log K$.

Proof. For a fixed set of omics weights, the weighted loss for a cell is

$$\ell_\alpha(g,x) = \sum_{m=1}^{M} \alpha^m H(p_{g^m}(\cdot \mid x^m)). \tag{S12}$$

Because $\alpha^m \geq 0$ and $\sum_m \alpha^m = 1$, $\ell_\alpha(g,x)$ is bounded whenever the entropy loss is bounded. Applying a standard Rademacher-complexity generalization bound to the entropy-loss class gives, with probability at least $1-\delta$,

$$\mathbb{E}_{x\sim\mathcal{X}}[\ell_\alpha(g,x)] \leq \frac{1}{N}\sum_{i=1}^{N} \ell_\alpha(g,x_i) + \hat{\mathfrak{R}}_N(\mathcal{G}) + \sqrt{\frac{\ln(2/\delta)}{2N}}. \tag{S13}$$

Expanding ℓ_α over the M omics layers gives

$$\mathbb{E}_{x\sim\mathcal{X}}[\ell_\alpha(g,x)] \leq \frac{1}{N}\sum_{i=1}^{N}\sum_{m=1}^{M} \alpha_i^m H(p_{g^m}(\cdot \mid x_i^m)) + \hat{\mathfrak{R}}_N(\mathcal{G}) + \sqrt{\frac{\ln(2/\delta)}{2N}}. \tag{S14}$$

Writing the empirical weighted term compactly as $\sum_m \mathbb{E}[\alpha^m \hat{H}(g^m, x^m)]$ yields Eq. (S11). $\square$

Theorem 1 indicates that, for a fixed architecture, reducing the empirical weighted entropy term tightens the controllable part of the GR upper bound. This motivates uncertainty-aware cell-wise omics weighting.

In Eq. (S11), the omics weights are intended to be cell-dependent, and the compact notation $\sum_{m=1}^{M} \mathbb{E}[\alpha^m \hat{H}(g^m, x^m)]$ corresponds empirically to

$$\frac{1}{N}\sum_{i=1}^{N}\sum_{m=1}^{M} \alpha_i^m H_i^m, \tag{S15}$$

where $\boldsymbol{\alpha}_i = (\alpha_i^1, \ldots, \alpha_i^M) \in \Delta^{M-1}$ denotes the omics-weight vector for cell i. Since the simplex constraint is imposed independently for each cell, the weighted empirical-risk term is separable across cells:

$$\min_{\{\boldsymbol{\alpha}_i\}_{i=1}^{N}} \frac{1}{N}\sum_{i=1}^{N}\sum_{m=1}^{M} \alpha_i^m H_i^m = \frac{1}{N}\sum_{i=1}^{N} \min_{\boldsymbol{\alpha}_i \in \Delta^{M-1}} \sum_{m=1}^{M} \alpha_i^m H_i^m. \tag{S16}$$

Therefore, for a fixed architecture, minimizing the controllable empirical term of the GR bound can be performed independently for each cell. To prevent the resulting weights from collapsing onto a single omics layer, we further introduce an entropy regularizer for each $\boldsymbol{\alpha}_i$. This leads to the uncertainty-aware cell-wise weighting formulation derived in the following subsection.

S1.4 Risk-guided derivation of uncertainty-aware fusion weights

The main text uses entropy-based RU to compute cell-wise omics weights for uncertainty-aware fusion. For cell i, the controllable part of the GR upper bound motivates the following optimization problem:

$$\min_{\boldsymbol{\alpha}_i \in \Delta^{M-1}} \sum_{m=1}^{M} \alpha_i^m H_i^m + \frac{1}{\lambda}\sum_{m=1}^{M} \alpha_i^m \log \alpha_i^m, \quad \lambda > 0, \tag{S17}$$

where Δ^{M-1} is the probability simplex. The first term down-weights high-uncertainty omics evidence, while the entropy regularizer prevents omics collapse.

Using a Lagrange multiplier ν for the simplex constraint, the Lagrangian is

$$\mathcal{J}(\boldsymbol{\alpha}_i, \nu) = \sum_{m=1}^{M} \alpha_i^m H_i^m + \frac{1}{\lambda} \sum_{m=1}^{M} \alpha_i^m \log \alpha_i^m + \nu \left(\sum_{m=1}^{M} \alpha_i^m - 1 \right). \tag{S18}$$

Setting $\partial \mathcal{J} / \partial \alpha_i^m = 0$ gives

$$H_i^m + \frac{1}{\lambda} (\log \alpha_i^m + 1) + \nu = 0, \tag{S19}$$

and therefore

$$\alpha_i^m = C_i \exp(-\lambda H_i^m). \tag{S20}$$

The normalization constant C_i is determined by the simplex constraint, yielding the closed-form Gibbs-type uncertainty-aware fusion weight,

$$\alpha_{i,\text{ent}}^m = \frac{\exp(-\lambda H_i^m)}{\sum_{m'=1}^{M} \exp(-\lambda H_i^{m'})}. \tag{S21}$$

Thus, omics representations with lower RU receive larger weights, whereas ambiguous or noisy omics signals are down-weighted.

S2 Additional quantitative results

S2.1 AMI comparison on benchmark datasets

The main text reports ARI and NMI for the state-of-the-art comparison. The corresponding AMI results are provided in Supplementary Table S1.

Table S1: Comparison of clustering performance on ten datasets, with average AMI values over five runs are reported. The best is in bold, and the second-best is underlined.

Method	MA-53	MA-54	MA-55	MA-56	Human cell line mixture	Mouse brain	Specter PBMC	BREM-SC PBMC	BMNC	SLN111
	AMI	AMI	AMI	AMI	AMI	AMI	AMI	AMI	AMI	AMI
DealMVC	0.3328	0.2951	0.2833	0.2596	0.7790	0.2407	0.6155	0.4568	0.7529	0.5455
scDRMAE	<u>0.6375</u>	<u>0.5470</u>	<u>0.5562</u>	<u>0.5389</u>	<u>0.9325</u>	<u>0.6118</u>	0.6736	<u>0.9114</u>	0.7383	0.5571
scEMC	0.1774	0.0417	0.0423	0.1455	0.7521	0.4716	**0.7300**	0.8606	0.6714	**0.6079**
scMCs	0.5297	0.4071	0.4471	0.4513	0.9032	0.4384	0.3786	0.6584	0.4726	0.3371
MoClust	0.4633	0.4939	0.4757	0.3913	0.0494	0.2114	0.5876	0.6138	0.6413	0.4630
CVCL	0.2060	0.2167	0.2022	0.2356	0.4848	0.1409	0.6322	–	0.2253	0.1292
scMVC	0.4363	0.3079	0.3028	0.3420	0.3543	0.5086	0.6037	0.8677	0.7477	0.4919
MCMC	0.3438	0.3423	0.3284	0.4152	0.3765	0.2869	0.616	0.659	0.3736	0.5445
MGCMVC	0.3538	0.2836	0.3465	0.3175	0.6181	0.2733	0.5963	0.8653	<u>0.7588</u>	0.4984
scURL	**0.7644**	**0.6291**	**0.6356**	**0.6473**	**0.9364**	**0.6755**	<u>0.7131</u>	**0.9473**	**0.7854**	<u>0.5981</u>

"–" denotes that no results are available for the method on the corresponding dataset.

S2.2 AMI ablation results

The AMI results for the ablation study are provided in Supplementary Table S2. They further support the complementary effects of the multi-granular calibration module (MCM) and uncertainty-aware fusion.

Table S2: Ablation studies of the key components. Mean AMI values over five runs are reported.

MCM	UAF	MA-53	MA-54	MA-55	MA-56	Human cell line	Mouse brain	Specter PBMC	BREM-SC PBMC	BMNC	SLN111
		AMI	AMI	AMI	AMI	AMI	AMI	AMI	AMI	AMI	AMI
×	×	0.7205	0.5880	0.6015	0.6070	0.8575	0.6025	0.6997	0.8863	0.7330	0.5403
✓	×	0.7621	0.6163	0.6270	0.6473	0.8960	0.6139	0.7010	0.9238	0.7387	0.5627
×	✓	0.7574	0.6249	0.6279	0.6402	0.9039	0.6111	0.7020	0.9182	0.7725	0.5862
✓	✓	0.7644	0.6291	0.6356	0.6473	0.9364	0.6755	0.7131	0.9473	0.7854	0.5981

S3 Additional visualizations and sensitivity analyses

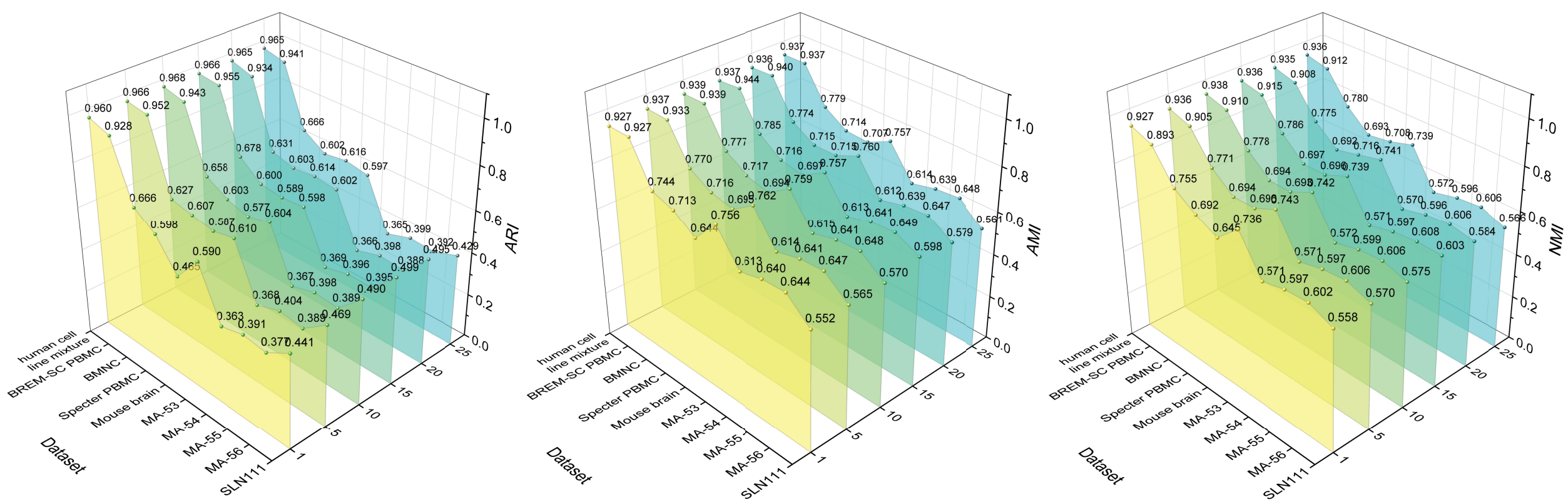

Figure S1: Sensitivity of the LSC neighborhood size C. scURL remains stable across a broad range of C values, especially when $5 \leq C \leq 25$.

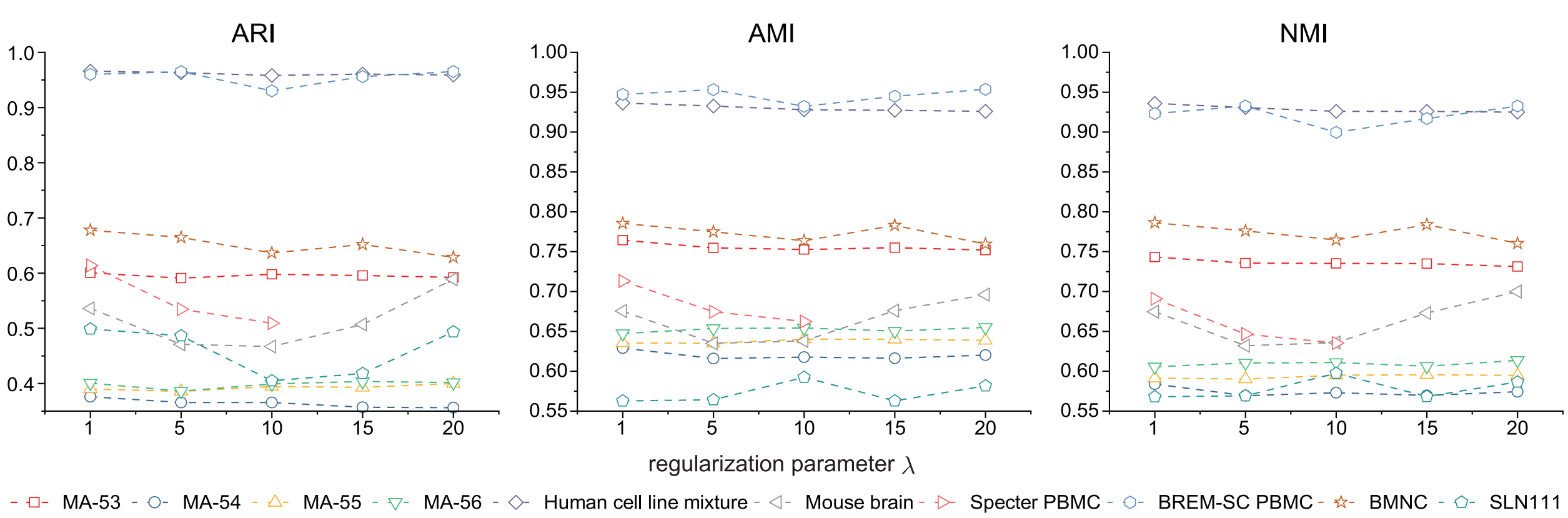

Figure S2: Sensitivity of the uncertainty-aware fusion sharpness parameter λ. scURL remains stable under moderate changes in λ.

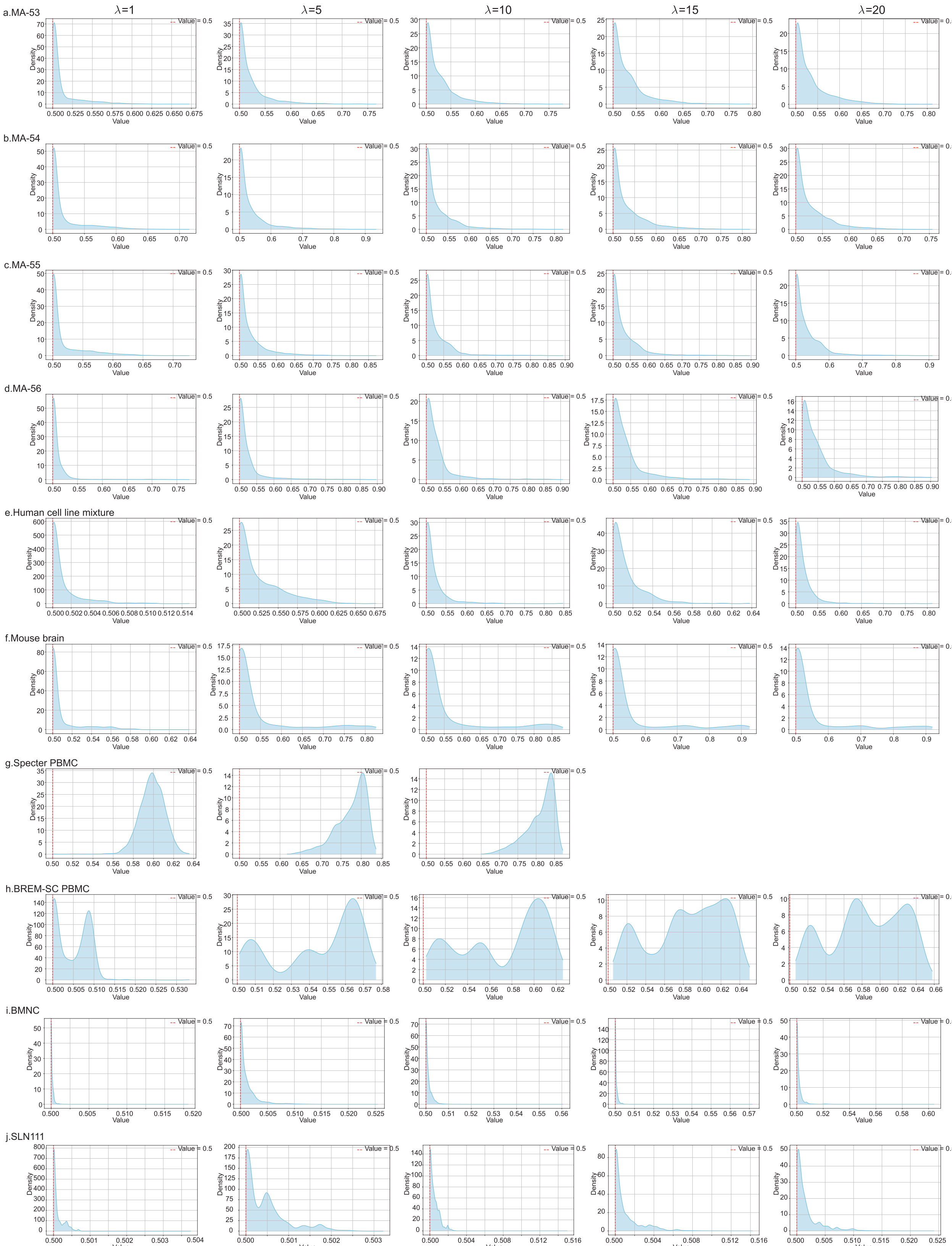

Figure S3: Cell-wise ℓ_2 norm squared distributions of uncertainty-aware fusion weight vectors under different values of λ. For each cell, $\|\boldsymbol{\alpha}_i\|_2^2 = \sum_m (\alpha_i^m)^2$ reflects the sharpness of the omics-weight distribution. Smaller values indicate more balanced omics contributions, whereas larger values indicate sharper weighting toward lower-uncertainty omics layers.
